# Influence of smartphone-based physical activity intervention on executive functions and cardiometabolic disease risk in obese young adults: a pilot randomised controlled trial

**DOI:** 10.1007/s40200-023-01182-9

**Published:** 2023-01-13

**Authors:** Sapna Vithoba Domal, Baskaran Chandrasekaran, Hari Prakash Palanisamy

**Affiliations:** 1 Department of Exercise and Sport Sciences, Manipal College of Health Professions, Manipal Academy of Higher Education, Madhav Nagar, 576104 Manipal, Karnataka India; 2 Department of Speech and Hearing, Manipal College of Health Professions, Manipal Academy of Higher Education, 576104 Manipal, Karnataka India

**Keywords:** m-health, Obesity, Weight loss, Blood pressure, Executive control, Physical activity

## Abstract

**Purpose:**

Smartphone is an impending solution to influence long-term behavior change, including physical activity; however, the evidence regarding personalized prescription remains mixed in obese. We aimed to explore the influence of smartphone-based physical activity promotion on weight loss and cognitive functions in obese young adults.

**Methods:**

In our pilot randomised controlled trial, 24 obese adults were randomized to two conditions: (1) EXI group receiving a smartphone-application based personalized physical activity prescription; (2) CONT group continuing their routine work for eight weeks. Executive functions and cardiometabolic risk variables [body and fat mass, waist circumference (WC), blood pressure (BP)] and executive functions were measured before and after eight weeks.

**Results:**

Our study findings revealed a significant improvement in reaction times with overall (H = 7.71, p = 0.005), congruent stimuli (H = 4.43, p = 0.03) and incongruent stimuli (H = 5.35, p = 0.02) between groups. Though EXI participants reduced their fat mass by 5.07 kg more than CONT group users after eight weeks, the findings were statistically insignificant. Similarly, our study did not find significant differences in body mass, WC, BP or accuracy between EXI and CONT groups. There was a decreased user engagement (H = 5.80, p = 0.564) after the 3rd week of the study period.

**Conclusion:**

Short-term smartphone-based physical activity programs may offer favourable cognitive benefits to young adults; however, the weight loss benefits remain unconvincing.

**Trial Registration:**

The study was registered prospectively with the Clinical Trials Registry of India (CTRI/2022/02/040202).

**Supplementary Information:**

The online version contains supplementary material available at 10.1007/s40200-023-01182-9.

## Introduction

Obesity is a growing public health crises in the modern urbanized society due to high-calorie foods, sedentary behaviours and low physical activity [1]. The altered energy balance has increased the risk of obesity, eventually leading to coronary artery diseases, cancer and cognitive disorders [2]. The deranged glucose and lipid metabolism, insulin resistance, leptin-adiponectin resistance, low brain-derived neurotrophic factors and altered neurogenesis are putative mechanisms underpinning obesity and cognitive dysfunction [3]. Hence healthy lifestyle choices such as improving physical activity or reducing a high-calorie diet are promising measures to combat overweight and obesity by restoring the above physiological mechanisms. Optimizing weekly physical activity among obese might favourably reduce the cardiometabolic risk such as weight, body mass index (BMI), blood pressure (BP), peripheral fat content and eventually offer protection to cognitive functions, mainly executive functions and problem-solving skills [4, 5].

Despite the convincing evidence claiming the beneficial effects of physical activity, only a fraction of the global population meets the recommended weekly dose of physical activity [6, 7]. Improving people’s compliance with physical activity always remains a challenge for public health scientists [8]. Being ubiquitous, smartphones have become an integral part of contemporary men’s and women’s lives and have the potential to measure, intervene and influence individual’s everyday physical activity [9, 10]. Recent smartphone applications such as EXi claim to provide personalized physical activity targets and real-time feedback, which may alter behavior and improve long-term compliance to biological activity. However, the efficacy of such programs remains unexplored.

Through our pilot randomized controlled trial, we attempted to answer the two questions: (1) whether the smartphone-based application can alter the cardiometabolic risk factors (blood pressure, fat %, body mass) and executive functions (reaction times and accuracy) among overweight and obese adults in 8 weeks? (2) whether the smartphone-based application can alter the physical activity levels among overweight and obese adults in 8 weeks?

## Methods

We conducted a pilot randomized study in 14 obese men and women. We reported the findings as per the recommendations of CONSORT extension for randomized pilot and feasibility trials [11]. The checklist is attached as supplementary file S1. The present study started after approval from the Institutional Ethics Committee of a multifaceted university (IEC 740/ 2021). The trial was prospectively registered in the Clinical Trial Registry of India (CTRI/2022/02/040202). The study conformed to the ethical principles for human participants laid by the Declaration of Helsinki [12].

### Study design

In our pilot randomized controlled trial, we explored the short term efficacy (8 weeks) of the personalized physical activity approach through smartphone-based application among overweight and obese adults. The participants were randomized into two groups: (1) the Exercise Intervention (EXI) group received an app installed on their smartphones and guided for a graded step count challenge as chosen by the participants for the next eight weeks; (2) waitlist control (CONT) group participants continued their routine daily work and were asked not to alter their routine for next eight weeks. Supplementary file S2 shows the overall design of our randomized pilot trial.

### Participants

Potential participants aged 18–35 years, self-reporting to be overweight or obese, daily sitting time of more than six hours per day and physical activity levels of less than 150 min per week or less than 30 min of activity in a day, live in and around the university were invited to participate in the study. The participants were found eligible if the participants had confirmed overweight or obesity with a measured body mass index (BMI) of more than 23.0 kg/m^2^ and waist circumference (WC) in men (> 90 cm) and women (> 80 cm) [13, 14]. Further, the participants eligible for randomization to the EXI group should have an android based smartphone (over version 5.0 and above) and be willing to install the application provided by the investigators. Volunteers with established cardiovascular, musculoskeletal and neoplastic disorders were excluded from the study. Individuals with fewer than three weeks of musculoskeletal trauma or any psychological illness such as documented depression or anxiety disorders that limit adequate participation in physical activity were excluded from the study. All the volunteers provided written informed consent after the randomization.

Intervention.

We administered the physical activity intervention through a smartphone-based application named EXi. EXi is an evidence-based application approved by National Health Services, United Kingdom [15]. EXi aids in prescribing personalized physical activity programs based on the user’s health and fitness, which is automatically calculated based on self-reported BMI, age and the presence of chronic diseases. As the participants download the EXi app and enter their demographic, health and fitness data, the application creates a digital personalized physical exercise plan for the next 12 weeks. The users of the EXi app were instructed to meet an individualized graded target for each week via step count, which is tracked in the background via their smartphone (e.g. four days of 6,000 steps) (supplementary file S3a). EXi application creates a 12-week plan with the volume of exercise or step counts targets are graded each week based on targets met by the end-users. We have considered only the first eight weeks of tracking as our study protocol involved only short-term physical activity promotion for eight weeks. The EXi application tracks the end-user step count and the weekly steps target which were shared by the participants to the primary investigator every weekend for next eight weeks. The distance covered and intensity of activity achieved can be downloaded as a pdf file (Supplementary file S3b).

### Outcomes

The present pilot study explored the influence of physical activity on cardiometabolic disease risk factors (BMI, BP, WC, fat %) and executive functions.

#### Body mass index

Body height was measured in a wall-mounted stadiometer with participants standing, and feet pointed straight. The height was measured without the shoes to the nearest ≈ 1 cm. The weight was measured with the participants in light clothing and without shoes to the nearest ≈ 0.1 kg. BMI was estimated from the Quetelet’s index, derived from the ratio of the weight in kilograms divided by the square of the height in meters [16].

#### Blood pressure

BP measurements were taken in the seated position using a Diamond LED Deluxe BP apparatus (Meserve Medical Center, New Delhi, India) as per the recent recommendations from American Heart Association [17]. Each participant was instructed to be rested in a seated position for at least 10 min in an armed chair, both feet on the floor and arm supported at heart level before the measurement. The participant was asked to avoid caffeine, exercise and smoking for at least 30 min before measurement. An appropriately sized cuff (cuff bladder encircling at least 80% of the arm) was used to ensure accuracy. Highest of the three readings measured is recorded and analyzed further.

#### Fat percentage

The participants were instructed to be in minimal clothing for the skinfold measurement. The fat percentage was estimated from the sum of seven skinfolds using calibrated Lange callipers (Beta Technology Inc, Cambridge, Maryland) [18]. The calibration was done using a 25 mm calibration block before each individual’s measurement. The seven skinfolds used for fat percentage estimation were chest, triceps, abdominal, thigh, subscapular, suprailliac and midaxillary skinfolds. The skinfolds were marked with an indelible marker and measured using a calliper nearest to 0.1 mm at the right side of the body. If the differences between the two measurements were more than 10%, the third measurement was done, and the highest of the two closest skinfold thickness was recorded for the site. Body density was estimated using sex-specific equations of Jackson and Pollock et al. 1985 [19]: Men [1.112 − 0.00043499 (Σ7SF) + 0.00000055 (Σ7SF)^2^−0.00028826 (age)]; women [1.097–0.00046971 (Σ7SF) + 0.00000056 (Σ7SF)^2^-0.00012828 (age)] where Σ7SF is the sum of 7 skinfolds in mm. Fat percentage was then estimated from Sir’s equation [%BF=(495/density)–450], where BF – body fat, thereby fat and fat-free mass were derived from the fat percentages considering the body mass of the participants [20, 21]. A postgraduate with experience of two years in estimating fat percentage from skinfold measurement administered the tests.

#### Waist circumference

With participants standing in a relaxed position and arms side of the body, the narrowest part of the torso between ribs and upper portion of the hip bone using Gulick retractable measuring tape. The measurement was repeated twice, and the highest value was considered for analysis [21].

#### Executive functions

The executive functions were measured using the computer based Eriksen flanker test. All the congruent and incongruent stimuli (arrows) were presented on a laptop using inquisit software (version 6.5.2, Millisecond, Seattle, WA, USA) [22]. The participants were seated 50 cm away from the screen and presented with five arrows with two flanker arrows on either side of the target arrow, pointing the same side (congruent) or opposite direction (incongruent) of the target arrow in the centre. The participants were instructed to press the “p” or “q” keys if the target arrow in the centre is directed left or right, respectively. The appearance of arrows was preceded by a ‘+’ symbol in the centre of the screen, indicating the subsequent target stimuli occur in the position. Thirty-two trials were presented with eight practice trials and 24 test trials. Each trial was presented for a maximum of 1750 ms (the target and distractors were presented for a maximum of 1000 ms and timeout for 750 ms). Once the participants completed the assigned task, the summary and raw files were automatically saved as inquisit files and could be retrieved as excel files. The accuracy and reaction times for overall, congruent and incongruent stimuli were calculated and stored by the software, later retrieved for analysis.

### Sample size

The sample size was estimated using the upper confidence limits (UCL) as outlined by Whitehead, 2015 [23]. To find a moderate effect (at least 20 milliseconds difference in reaction times) in intervention at an inflation factor of 1.40 UCL factor, we required a sample size of 16 at an 80% confidence level and a level of significance of 95% using the formula; nM = [(r + 1) (Z_1−β_+Z_1−α_/2)^2^ s^2^ UCL]/rd^2^ where UCL is upper confidence limits and d is the estimated effect size [23]. Allowing for 20% drop-out, we required 19 participants to check the effectiveness of a short-term physical activity promotion through wearable.

### Randomization and allocation concealment

A statistician blinded to the study randomized the participants to the EXI and CONT groups using computer-generated randomization (https://www.randomizer.org/). Two sets of numbers were generated with seven unique numbers in a set, with each number being unique per set. Once two sets of seven random numbers were obtained, they were sealed in opaque envelopes, and the participants were assigned interventions at the initial screening visit.

### Blinding

An independent researcher who administered the cognitive tests did not know about the randomization or the interventions employed in the study. However, the primary investigator who measured the cardiometabolic risk variables (BP, body composition and fat percentage) was not blinded to the randomization. The participants knew their randomized order and the interventions.

### Procedure

The potential participants visited the laboratory twice during the study period: (1) familiarisation and baseline measurement visit: All the participants visited the temperature and humidity-controlled laboratory between 7:00–8:00 AM in a fasted state for 12 h and adequate sleep of 8 h. The participants were familiarised with the baseline variables measurements such as cardiometabolic risk (BP, WC, skinfold measures) and executive functions measurements. Once the participants were familiarised, the participants were given a standard breakfast (consisting of 33% of total daily intake). Immediately after, the participants were instructed to rest in a seated position for 15 min, and baseline measurements were administered. After the baseline assessments, the participants picked up the sealed envelopes and were allocated the assigned interventions: EXI or CONT. The EXi group participants were instructed to install the EXi app, familiarised themselves with the baseline data entry into the app, set the weekly target step count and send the weekly progress to the primary investigator for the next eight weeks. The participants of the CONT group were instructed to continue their routine work and to restrain from any new exercise or physical activity regime; (2) post-study period: after eight weeks of the study period, the participants returned to the laboratory again between 7:00–8:00 AM in a fasted state and the baseline measures such as cardiometabolic disease risk and executive functions were repeated. Except for the computer-based executive function measurement, other outcome measurements such as BP, WC and fat percentages were not blinded.

### Data analysis

The statistical analyses for the present pilot randomized controlled trial were performed using JASP statistical software version 0.16.3.0 (University of Amsterdam, Germany). The continuous variables (cardiometabolic risk and cognitive measures) at baseline were compared using Mann Whitney U test. Wilcoxon signed-rank tests were performed to evaluate the intervention effect (within-group) by time from baseline (t_0_) to post-intervention (t_8_). Kruskal-Wallis tests were conducted to evaluate the between-group differences for EXI and CONT groups on all outcomes’ delta (Δ) scores (post-intervention score – baseline score). Post hoc Tuckey’s correction was carried out when significant between-group differences were revealed for all measures. Intention to treat analysis was followed with the analysis of all randomised participants inspite of lost to followup. The statistically significant level was set at *p* < 0.05 (two-tailed).

## Results

**Fig. 1:**
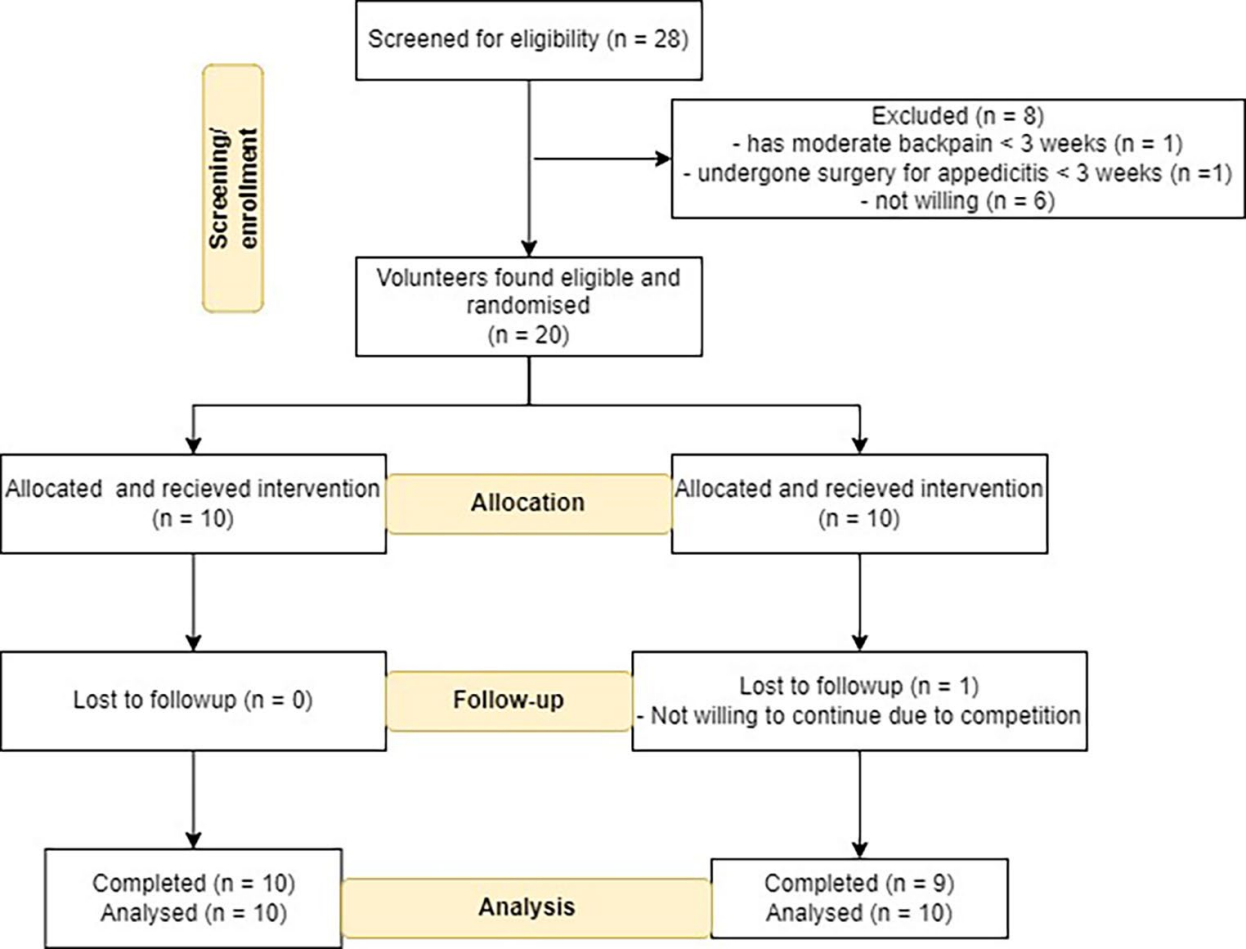
Flow diagram depicting the screening, inclusion of the participants

Though 24 potential participants were initially contacted, 20 (83%) were found eligible for the eligibility criteria and randomised to EXi group (n = 10) and control group (n = 10). Majority of the participants (n = 19; 95%) completed the eight weeks of the study. One participant from the EXi group was not able to continue due to competition at the end of 5th week. Finally, nine participants (90%) from the EXi group and ten participants (100%) from the CONT group completed the study. The study participants were recruited between February 2022 – May 2022. The participant’s screening, eligibility, inclusion and analysis are shown in Fig. 1.

### Baseline characteristics

The baseline characteristics of the demographics, cardiometabolic risk factors and executive function variables are depicted in Table 1. Twelve males and seven females completed the study. The majority of the participants (n = 15; 75%) were obese class I, while the remaining (n = 5; 25%) were obese class II according to the BMI standards of WHO-Asia classification [24]. Mann Whitney U tests showed that all the baseline cardiometabolic risk and executive function variables were not statistically different among EXI and CONT groups except overall and incongruent reaction times.

**Table 1:** Baseline characteristics of the participants

Variable	Overall (n = 20)^Ψ^	EXI group (n = 10)^Ψ^	CONT group (n = 10)^Ψ^	W	p
**Age (yrs)**	24 (4.5)	24 (3)	27 (11)	14.00	0.748
**Gender**	13 M (65%), 7 F (35%)	7 M (70%), 3 F (30%)	6 M (60%), 4 F (40%)	0.28^#^	0.598
**Height (cm)**	167.55 (16.85)	163.30 (15.46)	169.35 (8.60)	15.00	0.610
**Weight (kgs)**	76.36 (17.84)	70.41 (14.94)	82.38 (11.78)	17.00	0.352
**BMI**	28.40 (2.13)	27.40 (2.60)	28.40 (0.65)	15.00	0.589
**SBP**	123.50 (4.50)	123.50 (3.00)	124.00 (12.00)	12.50	1.000
**DBP**	66.00 (9.50)	67.00 (10.50)	65.00 (7.00)	9.50	0.665
**Fat (%)**	25.96 (17.45)	25.96 (19.13)	32.98 (15.18)	13.00	0.914
**Fat mass (kgs)**	22.07 (6.99)	20.51 (9.69)	24.43 (6.09)	18.00	0.257
**FFM (kgs)**	55.37 (21.39)	55.37 (17.79)	55.42 (20.44)	14.00	0.762
**WC (cms)**	95.85 (7.64)	93.72 (7.94)	98.90 (6.08)	16.00	0.476
**Overall accuracy (%)**	98.00 (8.00)	100.00 (6.00)	94.00 (12.25)	7.00	0.299
**Overall RT (ms)**	411.73 (68.11)	385.31 (55.27)	455.47 (32.46)	23.00	0.019*
**Congruent accuracy (%)**	100.00 (0.00)	100 (0.00)	100 (0.00)	Variance 0 after grouping	
**Congruent RT (ms)**	373.79 (77.37)	353.71 (50.44)	422.45 (57.83)	20.00	0.114
**Incongruent accuracy (%)**	96.00 (17.00)	100.00 (12.75)	76.50 (42.00)	6.00	0.209
**Incongruent RT (ms)**	447.17 (74.78)	388.73 (50.44)	507.94 (126.49)	23.00	0.019*

Ψ = given as median (interquartile range); # = Fischer exact test (X^2^); *p < 0.05; Abbreviations: BMI = body mas index, DBP = diastolic blood pressure, FFM = fat free mass, RT = reaction times, SBP = systolic blood pressure, WC = waist circumference

### Personalized physical activity participation

When treated with the Kruskal Wallis test, neither the weekly step count (H = 5.76, p = 0.564) nor the daily step count (H = 5.80, p = 0.564) was statistically different among 1st, 2nd, 3rd, 4th, 5th, 6th, 7th and 8th weeks of smartphone-based physical activity promotion. Though Tuckey’s post hoc analysis revealed an increase of 3052 steps from the baseline in EXI groups at the end of 3rd week, the improvement in step counts reduced after the 3rd week of EXI intervention (supplementary file S4).

### Physical activity on cardiometabolic disease risk

**Fig. 2:**
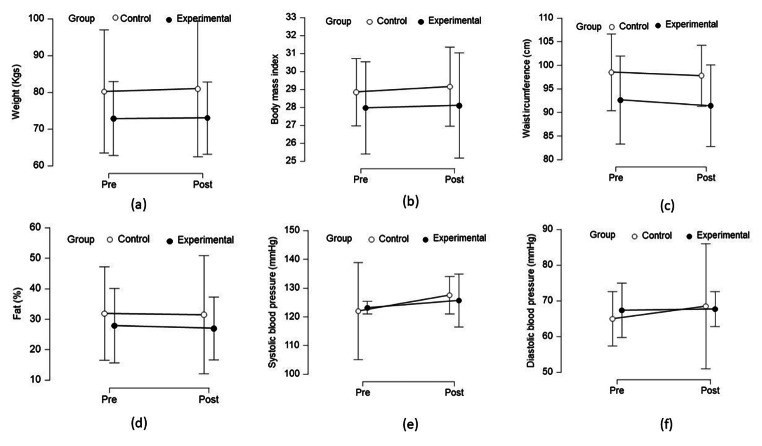
Differences in cardiometabolic risk variables: (a) weight, (b) body mass index, (c) waist circumference, (d) fat (%), (e) systolic blood pressure, (f) diastolic blood pressure within and between groups (EXI and CONT).

Table 2 shows the statistical differences in the cardiometabolic risk variables (weight, BMI, WC, SBP, DBP, fat%, fat mass and fat-free mass) within and between the groups. Figure 2 (a – f) revealed the non-significant differences in cardiometabolic risk variables within and between the groups.

**Table 2:** Mean ± Standard Deviation of all variables of EXi and control groups by using Kruskal Wallis test

Variables		EXi group (n = 10)			Control group (n = 10)			Δ_e_ - Δ_c_		Within group	Between the groups	
		Pre (t_0_)	Post (t_8_)	Δ_e_ (t_8_ - t_0_)	Pre (t_0_)	Post (t_8_)	Δ_c_ (t_8_ - t_0_)		H	p	H	p
**Weight (kgs)**		72.86 ± 9.6	73.02 ± 9.35	0.16 ± 5.53	80.24 ± 10.52	80.95 ± 11.59	0.71 ± 7.144	-7.65 ± 4.611	0.006	0.94	2.881	0.09
**BMI**		27.96 ± 2.44	28.10 ± 2.79	0.03 ± 1.28	28.28 ± 1.18	29.15 ± 1.38	0.30 ± 1.56	-0.96 ± 1.01	0.03	0.85	0.93	0.33
**WC (cm)**		92.63 ± 8.91	91.43 ± 8.25	-1.20 ± 4.24	98.52 ± 5.09	97.82 ± 4.06	-0.70 ± 5.20	-6.13 ± 3.35	0.28	0.59	3.14	0.07
**SBP (mmHg)**		123.16 ± 2.13	125.66 ± 8.733	2.50 ± 4.06	122 ± 10.58	127.5 ± 4.12	5.50 ± 4.97	-0.33 ± 3.21	2.09	0.14	0.05	0.81
**DBP (mmHg)**		67.33 ± 7.23	67.66 ± 4.63	0.33 ± 4.08	65 ± 4.76	68.50 ± 11.	3.5 ± 4.99	0.75 ± 3.22	0.47	0.49	0.09	0.75
**Fat (%)**		27.91 ± 11.62	26.94 ± 9.77	-0.96 ± 6.25	31.84 ± 9.59	31.48 ± 12.19	-0.36 ± 7.65	-4.23 ± 4.94	0.001	0.96	0.61	0.43
**Fat Mass (Kgs)**		19.79 ± 6.76	19.46 ± 6.14	-0.33 ± 3.66	24.88 ± 5.24	24.51 ± 6.93	-0.37 ± 4.48	-5.07 ± 2.89	0.09	0.76	3.42	0.06
**Fat Free Mass (Kgs)**		53.05 ± 13.50	53.41 ± 11.02	0.35 ± 7.96	55.31 ± 14.42	56.43 ± 17.36	1.11 ± 9.76	2.64 ± 6.30	0.006	0.94	0.29	0.58
**Overall**	**Accuracy (%)**	97.33 ± 4.13	95.13 ± 3.13	-2.19 ± 4.55	87.75 ± 16.82	98.95 ± 2.08	11.20 ± 5.58	2.88 ± 3.60	0.02	0.87	0.20	0.65
	**Reaction Time (ms)**	379.20 ± 45.53	357.69 ± 34.48	-21.21 ± 34.53	458.89 ± 41.46	444.34 ± 109.21	-14.55 ± 42.29	-83.16 ± 27.30	1.46	0.22	7.71	0.005^**^
**Congruent**	**Accuracy (%)**	*The variance is equal to 0 after grouping on time, group*										
	**Reaction Time (ms)**	354.46 ± 46.18	344.42 ± 40.33	-10.04 ± 38.22	419.26 ± 48.66	421.65 ± 121.40	2.09 ± 46.81	-70.85 ± 30.21	0.69	0.40	4.33	0.03*
**Incongruent**	**Accuracy (%)**	94.33 ± 8.77	81.83 ± 24.99	-12.50 ± 12.11	69.50 ± 34.10	98.0 ± 4.0	28.50 ± 14.84	4.33 ± 9.57	0.02	0.87	0.02	0.87
	**Reaction Time (ms)**	395.29 ± 47.55	372.12 ± 30.89	-23.17 ± 41.73	532.28 ± 100.63	449.47 ± 111.30	-82.81 ± 51.12	-107.17 ± 32.99	1.46	0.22	5.35	0.02*

Abbreviations: BMI = Body Mass Index, DBP = diastolic blood pressure, EXi = the group received smartphone based physical activity intervention, SBP = systolic blood pressure, t_0_ = baseline measure, t_8_ = 8th week measure, WC = waist circumference, * p < 0.05, **p < 0.01

#### Weight loss

We did not find any significant difference in weight, BMI, or WC within and between the groups (EXI and CONT). Besides, changes in fat%, fat mass and fat-free mass were also not significantly different within and between groups. Though the participants of the EXI group showed a weight loss of -7.65 kg and BMI change of -0.96 kg/m^2^, the findings did not reach statistical significance. Similarly, EXI participants showed a reduction of 4.23% in fat%, 5.07 kg in fat mass and an increase of 2.64 kg of FFM compared to control group. However, the study findings did not reach statistical significance.

#### Blood pressure

Mean SBP was found to reduce by 0.33 mmHg and while DBP increased by 0.75 mmHg in the participants of the EXI group compared to CONT group participants. However, the Kruskal Wallis test did not reveal significant differences in SBP and DBP within and between groups.

### Physical activity on executive functions

**Fig. 3:**
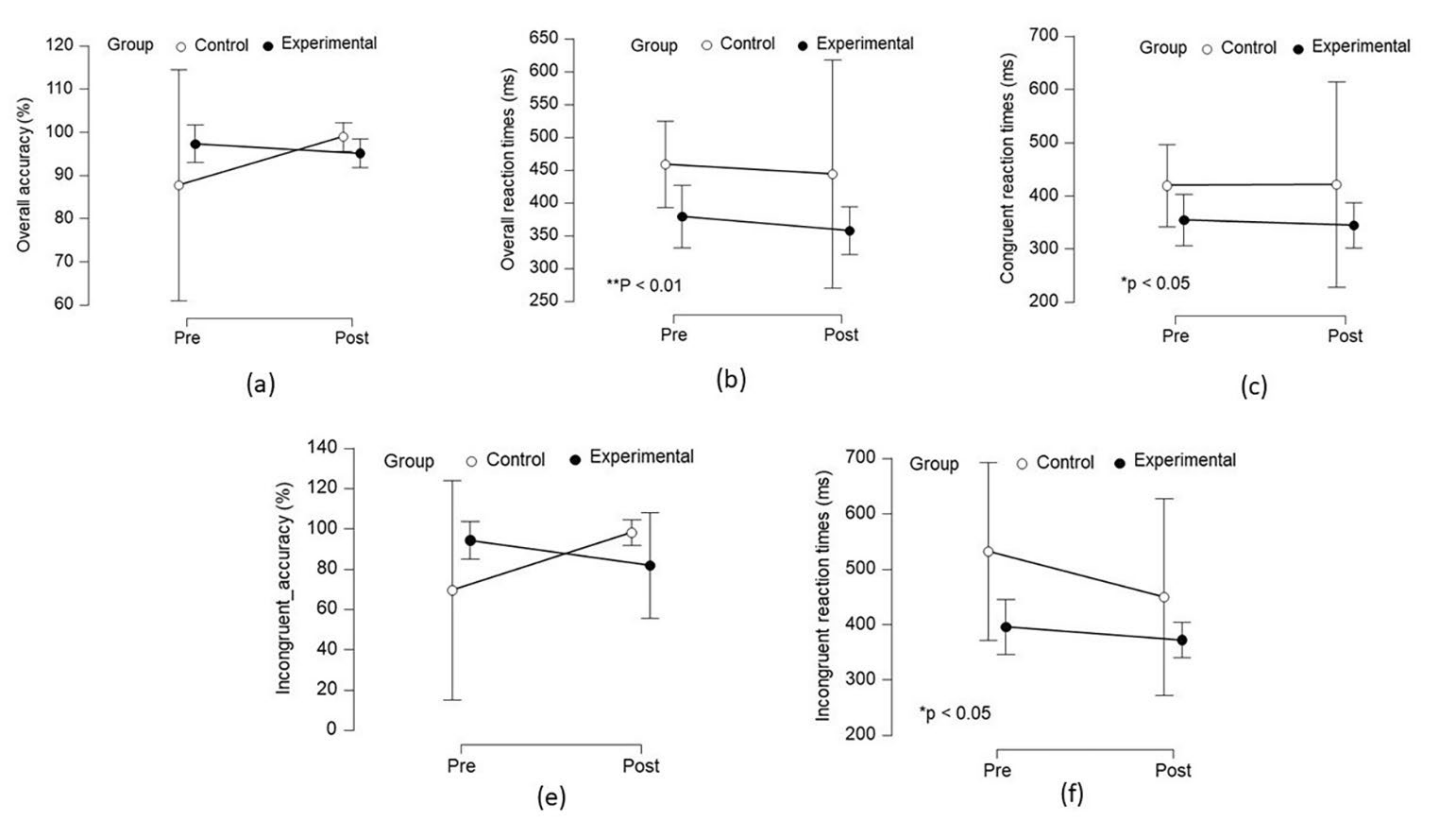
Outcome measures of Eriksen flanker test (Experimental and control group) - Descriptive Plots of the main effect within time and between group and interaction effect of time and group (a) Overall Accuracy (b) Overall Reaction Time (c) Congruent Reaction Time (d) Incongruent Accuracy (e) Incongruent Reaction Time

Table 2; Fig. 3 shows the differences in the reaction times and accuracy among EXI and CONT groups.

#### Reaction times

Our study findings revealed a significant difference in overall (H = 7.71, p = 0.005), congruent stimuli (H = 4.43, p = 0.03) and incongruent stimuli (H = 5.35, p = 0.02) between groups. We found a significant reduction of − 83 ms, − 70 ms, and − 107 ms with overall, congruent and incongruent stimuli.

#### Accuracy

Our study did not find any significant difference in overall accuracy (H = 0.20, p = 0.65) and incongruent stimuli (H = 0.02, p = 0.87) between groups. Similarly, the Kruskal Wallis test showed no significant differences in the accuracy with the overall, congruent and non-congruent stimuli within the groups. Table 2; Fig. 3 (a, e) shows the non-significant differences in the accuracy within and between the groups.

### Adverse events and adherence to the physical activity program

None of the participants among the EXI and CONT groups had any adverse effects such as injury related to the study during the eight weeks of the study. All the participants of the EXI group adhered only till the 3rd week and started decaying (1231–3159 steps) till eight weeks.

## Discussion

Our study attempted to explore the short-term health effects of smartphone-based personalized physical activity promotion in sedentary obese adults. Our study showed the participants of the EXI group improved their reaction times however failed to show any significant changes in the cardiometabolic risk variables compared to CONT group participants.

### Cardiometabolic disease risk

Our study found no significant changes in weight, BMI, WC, fat percentage or fat mass with smartphone-based personalized physical activity programs. Our findings are contrary to the previous meta-analytic findings, favoring smartphone-based intervention’s efficacy, including diet and physical activity programs on weight loss in adults. Kim (2020) found a significant effect on weight loss (− 2.40 kg) while failing to find a significant change in the BMI from a pooled analysis of five randomized controlled trials with a total of 1830 participants [9]. However, the review by Kim (2019) pooled studies that administered smartphone applications for both diet and physical activity, whereas our study did not control or regulate the diet. Our study did not find any significant change in the weight loss parameters, probably due to the following reasons: (1) poor adherence to the step count challenges from the third week to the eighth week of the intervention. This limitation of poor adherence to smartphone-based programs is addressed in contemporary evidence [25, 26]; (2) diet was not controlled in our pilot study. Physical activity or diet alone interventions often fail to find favourable weight loss effects. In contrast, combined diet and physical activity interventions positively affect weight loss [27]. (3) Hawthorne effect on the participants, including the CONT group, also might have altered the behaviour (increased physical activity) based on the known altered baseline cardiometabolic risk variables and feeling monitored for the next eight weeks [28]; (4) physical activity target was monitored from the smartphone-based application (EXi) while the validity of the step count with the app is relatively unknown [29]. (5) previous physical activity was not considered while this might have caused the insignificant differences in cardiometabolic risk among EXI and CONT groups.

Further, our study did not find any statistically significant reduction in SBP or DBP with the short-term smartphone-based PA promotion in obese adults. We found a reduction in SBP of − 0.33 mmHg, which is much lesser than pooled analysis of a recent metanalysis which reported a reduction of -2.28 mmHg with smartphone-based intervention in hypertensive patients [30]. However, the review involved smartphone-based diet, medication adherence, and physical activity [30]. Long-term studies have yet to explore the isolated effects of smartphone-based physical activity promotion on blood pressure.

### Executive functions

We found increased reaction times in the EXI group, while no significant differences in accuracy were found between EXI and CONT groups. Increased physical activity may improve the cortical circulation, muscle tone and preparedness, regulate leptin, cortisol and brain-derived neurotrophic factor, neural synapsis and potentiation that might have favorable effects on reaction times among obese adults [31, 32]. However, this favorable effect is not reflected in accuracy point of view as baseline accuracy was already high among both groups. Further, we must acknowledge significant baseline differences in the reaction times between EXI and CONT groups at the beginning of the study.

### Adherence to m-health intervention

We found a behavioral decay with our smartphone-based physical activity promotion, especially from the 3rd week of intervention in the end-users of the EXI group. The potential reasons for our study’s poor compliance or compliance decay could be the lack of gamification and social networking features in the EXi application administered in our study [33, 34]. Though the step count increased by 66% from the baselines in the third week, the daily step count deteriorated and was maintained at 33% higher than the baseline. Still, no statistically significant differences were observed between the weekly or daily step count across eight weeks.

Few limitations are worth mentioning in our pilot trial: (1) EXI application was not validated against any triaxial accelerometer for the step count; (2) the present study is a pilot design which might hinder the study findings from being adequately generalized. We recommend future randomized controlled trials to explore the effects of personalized physical activity participation through wearables among the obese population; (3) we did not consider the dietary intake of the subjects, which might have confounded our study results; (4) Since the present trial is pilot designed, we reckon future large sample trials to estimate them-health effectiveness on weight loss and cognitive functions.

To conclude, eight weeks of physical activity through wearable may have favorable effects on cognitive functions; however, the efficacy over cardiometabolic risk factors remains uncertain. We recommend adequately powered randomized controlled trials to explore wearable-based physical activity promotion’s effectiveness on cardiometabolic disease risk.

## Electronic supplementary material

Below is the link to the electronic supplementary material.

Supplementary Material 1

## Data Availability

All the available data was presented in the study. The main data will be available on reasonable request to the corresponding author. After the corresponding author gets the approval of other authors, he will be sharing to the requesting party with reservations.
